# Building a People-Centred Integrated Care Model in Urban China: A Qualitative Study of the Health Reform in Luohu

**DOI:** 10.5334/ijic.4673

**Published:** 2020-03-16

**Authors:** Di Liang, Lin Mei, Yingyao Chen, Ping Zhou, Xiaoguang Yang, Jiayan Huang

**Affiliations:** 1 School of Public Health, Fudan University, Key Lab of Health Technology Assessment, National Health Commission, CN; 2 Huashan Hospital, CN

**Keywords:** delivery system reform, people-centred integrated care, integrated service delivery networks

## Abstract

**Introduction:**

China has adopted a people-centred integrated care model to reform its severely hospital-centric and fragmented delivery system. As a template of this model in urban China, the Luohu Hospital Group has generated considerable public and academic interest to scale it up.

**Methods:**

Guided by a policy triangle framework, this qualitative study explored the context, actors, content, and process of founding the Luohu Hospital Group. Three semi-structured interviews and five focus groups were conducted among 35 key informants. Content analysis was used to analyse the data.

**Results:**

The reform in Luohu took place in a competitive health care market, based on the comprehensive health reform in Shenzhen. Under the strong leadership of the district government, the reform adopted comprehensive strategies to strengthen primary care and care coordination, improve the quality and efficiency of health care delivery, and promote population health. The reform achieved a high level of organisational integration but was still in the process of fulfilling professional and clinical integration.

**Conclusions and discussion:**

The establishment of the Luohu Hospital Group transformed the originally fragmented delivery system into a tightly integrated service delivery networks. Though valuable lessons have been generated, the reform and its impacts require ongoing monitoring.

## Introduction

Many countries have experimented with integrated care models, to combat health care challenges which come along with the epidemiological transition and population ageing. High-income countries often saw the mismatch between an increasing burden of non-communicable diseases and health care systems which focused on hospital-based and episodic acute care [1,2]. Many low- and middle-income countries failed to develop effective and high-quality primary care, despite the proliferation of vertically funded disease-specific initiatives [3]. In both settings, integrated care was considered as a means to provide more efficient and coordinated care, as patients were often affected by conditions that were chronic and occurring in combination (e.g., diabetes and hypertension, HIV and tuberculosis) [4]. The World Health Organisation has, therefore, suggested all countries to adopt a Framework on Integrated People-Centred Health Services and provide a continuum of care from health promotion to rehabilitation throughout people’s life course [5].

As an upper-middle-income country, the health care system in China was faced with challenges as found in both high-income countries and low- and middle-income countries. Since China launched a comprehensive health reform plan in 2009 [6,7,8], China has accomplished nearly universal insurance coverage, by covering more than 95% of citizens through three public health insurance programs [9]. However, China had a severely hospital-centric, fragmented, and inefficient delivery system [10].

Primary care facilities (e.g., community health centres in urban China, township health centres and village clinics in rural China) faced a shortage of qualified health workers and were not trusted by the public for its quality of care [10]. Providers at primary care facilities rarely acted as gatekeepers. Referrals were generally not necessary to see outpatient specialists. In addition, no mechanisms existed to coordinate services provided by primary care facilities and hospitals [11].

Public hospitals, which delivered the majority of outpatient and inpatient services in China, suffered from distorted provider incentives and poor governance structure [7,12,13,14]. On the one hand, under limited government funding and the fee-for-service system, physicians were incentivized to overprescribe drugs and overuse diagnostic tests, leading to rapidly rising costs and substantial waste [13,15]. On the other hand, governed by multiple administrative agencies which may issue rules contradictory in purposes and goals, public hospitals were left with little autonomy (e.g., autonomy in human resource management) to respond to health care market demands [7].

In 2016, the report “Deepening Health Reform in China: Building High-Quality and Value-Based Service Delivery”, published by the World Health Organization, World Bank, and the central government (Ministry of Finance, National Health and Family Planning Commission, and Ministry of Human Resources and Social Security), proposed to reform China’s delivery system in accordance with a People-Centred Integrated Care model [16]. Integrated service delivery networks (ISDN) were viewed as a pragmatic strategy to achieve this goal. The central government has issued a series of guidelines to promote and regulate ISDN [17,18,19,20,21,22].

One of the first and most well-known ISDN was Luohu Hospital Group in Shenzhen Municipality. As an urban district of Shenzhen, Luohu had one million population, and it’s per capita Gross Domestic Product was $31,517 in 2017 [23]. Luohu Hospital Group was established in August 2015 after integrating five district-level public hospitals and 23 public community health centres [24,25,26]. By July 2017, Luohu Hospital Group had more than 575 thousand registered clients [25]. The Luohu model was viewed as the template for building a people-centred integrated care model in urban China [25]. The Luohu model involved fundamental changes in the governance structure, payment method, care delivery model, and organisational culture. It was widely believed to have the potential to strengthen primary care and care coordination, to improve the quality and efficiency of health care delivery, and to promote population health. Early appraisals of its impact suggested a high level of satisfaction among patients and providers [27,28]. The Luohu model was endorsed by Prime Minister Keqiang Li and the National Health and Family Planning Commission [29].

Previous studies focus on the contents of the reform in Luohu. Little was known about how key stakeholders formed and implemented relevant policies in their context. As there are considerable public and academic interest in scaling up the Luohu model in other parts of China [30], it is crucial to understand how the reform took place; and what can be learned from this case to implement in a similar manner. The purpose of this study was, therefore, to examine the policy-making and implementation processes of the health reform in Luohu, including its policy environment and health care market context, the actors involved in the reform and their roles, and how the reform strategies were implemented to achieve integrated care.

## Methods

As external researchers evaluating Luohu Hospital Group independently for research purposes (without contract), we conducted three semi-structured interviews and five focus groups with 36 informants in total. Purposive sampling was used to identify 14 informants from the municipal government of Shenzhen, the district government of Luohu, and the Luohu Hospital Group, based on their expertise and their position. One focus group was conducted with six municipal government officials whose agencies were in involved in the health reform in Shenzhen (one from Municipal Health and Family Planning Commission which was responsible for the overall health reform, one from Municipal Establishment Office which oversaw the headcount quota system for health care facilities, one from Municipal Development and Reform Commission which set prices of medications and health services, one from Municipal Finance Bureau which financed public health care facilities, and two from Municipal Human Resources and Social Security Bureau which was in charge of health insurance programs). One government official from the District Health and Family Planning Commission of Luohu and eight hospital managers in the Luohu Hospital Group were interviewed, as they designed and/or implemented the health reform in Luohu. Another four focus groups were conducted with 21 health workers, nine from three hospitals and 12 from community health centres in the Luohu Hospital Group, who were randomly selected to share their experiences and perspectives. Individual interviews and focus groups, conducted in May 2017, were combined in qualitative studies to enhance data richness [31]. On average, interviews lasted for about one hour, and focus groups lasted for roughly two hours. All interviews and focus groups were conducted in a quiet space convenient to the informant. Informants were not compensated for their time. All interviews and focus groups were audio-taped and then transcribed into Word files.

Oral consent was also obtained for participants in the interview/focus group as well as for the audio recording. All interview materials were stored securely. Ethical approval was exempted from the authors’ review board because we were interested in the general information about establishing and managing the Luohu Hospital Group rather than informants’ personal experiences. Thus, no human subjects were involved.

**Figure 1:**
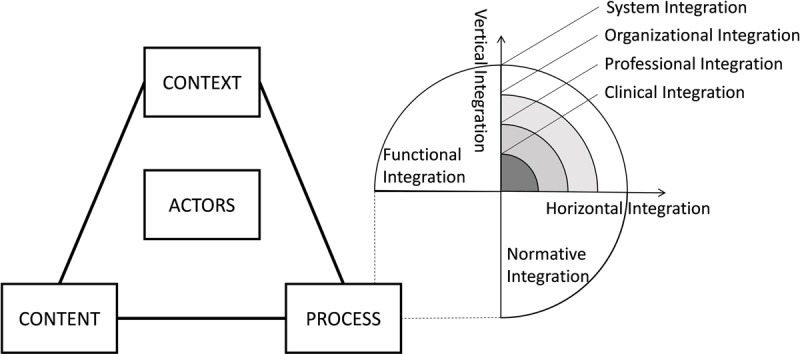
Conceptual framework. Note: The conceptual framework was built based on the policy triangle framework [32,33] and the framework of if integrated care [34,35].

As presented in Figure 1, the data analysis of this study was primarily guided by Walt and Gilson’s policy triangle framework, which was adapted according to the analysis of informants’ responses [32]. Grounded in a political economy perspective, the policy triangle framework has been widely used in health policy analyses in low- and middle-income countries [33]. This model not only focused on the content of reform (the “content” theme) but also emphasized the actors involved in the policy (the “actor” theme), processes of developing and implementing change (the “process” theme), and the context within which policy is developed (the “context” theme) [32]. We further augmented the “process” theme using the framework of integrated care developed by Valentijn and colleagues [34,35]. According to this model, integrating care involves integration processes at the macro-level (system integration), meso-level (organisational and professional integration), micro-level (clinical integration), and cross-level (functional and normative integration).

Content analysis was used to analyse the data. One researcher completed line-by-line open coding first, using gerunds to understand, examine, and compare data with data [36]. Gerunds were used as codes to emphasize actions of individuals and organizations of interest. After developing initial code lists, the code lists were then validated by another researcher. By grouping similar initial coding together, focus coding was developed and used to identify categories and themes under the policy triangle framework: context, actors, content, and process.

## Results

### Context

#### Health care financing

During the health reform over the last decade, Shenzhen achieved universal health insurance coverage through three public health insurance programs and was experimenting with multiple payment methods. Each health insurance plan covers approximately one-third of the population in Shenzhen, with Plan I the most comprehensive and flexible one. For outpatient care, beneficiaries of Plan I could choose any community health centre or hospital, while beneficiaries of Plan II/III could only go to one selected community health centre for first-contact care. For inpatient care, beneficiaries of Plan I/II could choose any hospital in Shenzhen, while beneficiaries of Plan III could only visit a designated hospital first. The municipal government hesitated to restrict beneficiaries’ choice of facilities or providers. This was partly because they were concerned about the pressure of public opinion, and they also explicitly valued beneficiaries’ ability to “vote with their feet.”

“It was easy to limit patients’ choice when you started (the health insurance program) from scratch. But now we are under a lot of public pressure and cannot limit patients’ choice anymore.” (a government official) The three health insurance programs in Shenzhen had two funding sources: medical savings accounts and health insurance funds (e.g., inpatient fund, outpatient fund). While medical savings accounts could only be used by their owners, health insurance funds were risk pools for all beneficiaries. The outpatient care for beneficiaries of Plan I was mainly paid by medical saving accounts under a fee-for-service scheme. The outpatient care for beneficiaries of Plan II/III was paid by the health insurance outpatient fund under a capitated model (about 13 yuan, 0.2% of the average monthly wage in Shenzhen). Inpatient care for all beneficiaries was paid by the health insurance inpatient fund under mixed schemes, including fee-for-service, service unit, per diem (daily rate), and diagnosis-related group system.

Shenzhen also reformed the government funding mechanisms for public health care facilities, including public hospitals and community health centres. Previously, public health care facilities received government funding based on the size of their staff with a quota. But the staff size with quotas was set by the government and could not meet facilities’ demand for human resources. As a result, public health care facilities had to rely on large numbers of contracted staff without a quota, whose salary was not subsidized by government funding. The lack of government funding drove public health care facilities to pursue profits instead of acting in the public interest. At present, the government funding for public hospitals and community health centres is delinked from the quota system.

“The central government has not increased the quotas for public hospitals in the past three years, while public hospitals were faced with enormous demands to expand their staff size.” (a government official).

#### Health care human resources

Many argued that human resource reform was urgently needed in public health care facilities. The quota system also left hospitals little autonomy to manage their workforce and made it difficult for community health centres to recruit and retain qualified general practitioners. For individual health workers, whether they were employed with quotas was linked to their job security and benefits (e.g., salary, pension, etc.). But only half of the health workers had a quota at many hospitals, and few health workers had a quota at community health centres. Contracted staff without a quota often worked more but had fewer benefits compared to quota staff. The municipal government intended to remove the headcount quota system in all public health care facilities but failed to do so. The municipal government perceived it as extremely difficult to reform the quota system themselves if having no national initiatives to follow. Hospital managers were concerned that having no quota made a position less attractive to prospective workers. Moreover, health workers with quotas were concerned about losing their current benefits and status. A promising next step would be to delink the quota system with health workers’ benefits, which might call for an increase in government funding.

#### Health care market

The health care market in Shenzhen was competitive as public health insurance programs had limited leverage in restricting patients’ choice of facilities or providers. As in other parts of China, beneficiaries of health insurance programs in Shenzhen could choose health care providers and facilities with little referral requirements. District-level hospitals (hospitals owned and governed by the district government) competed against city-level hospitals (hospitals owned and governed by the municipal government) which were often considered superior to district-level hosptials. Even community health centres affiliated to district-level hospitals had to compete against community health centre affiliated to city-level hospitals. The expansion of private health care facilities made the competition even fiercer. The health care market was particularly competitive in Luohu, where health care resources were highly concentrated. Thus, to survive the competition was considered one motivation for establishing the Luohu Hospital Group.

“The health care market in Luohu is extremely competitive. Our district-level hospitals can barely survive. We have to reform.” (a government official).

### Actors

#### The district government of Luohu

The district government established the Luohu Hospital Group by consolidating all district-level health care facilities in Luohu. The district government made a strong political commitment to provide supportive policies and financial arrangement in need. The party committee secretary told the director of the District Health and Family Planning Commission who was responsible for implementing the reform that he would be fulfill “whatever request you have made or have not made (to push the health reform forward).” The chief executive once said: “(I will) make you unable to find any excuses for failure in health reform.”

#### The municipal government of Shenzhen

The municipal government provided crucial support for the reform in Luohu. The bureau of labour and social security, which managed the three public health insurance programs in Shenzhen, developed a global budget model only for the Luohu Hospital Group.

But there were also tensions between reform initiatives in Luohu and those in Shenzhen. According to the health planning of the municipal government, future health care system in Shenzhen would mainly be composed of regional medical centres (i.e., primarily of city-level hospitals, most of which are tertiary hospitals) and primary care groups (i.e., district-level hospitals and community health centres). Regional medical centres would be responsible for providing advanced speciality care, conducting research, and training health workers. In primary care groups, family doctors at community health centres would be accountable for most basic health care needs in the community. The municipal government endorsed the Luohu Hospital Group as a breakthrough in forming a primary care group but were concerned about how to coordinate services provided by the Luohu Hospital Group and city-level hospitals as well as other district-level primary care groups.

It was noted that many challenges for the Luohu Hospital Group could not be addressed by itself or the district government. The health reform in Luohu required further support from the municipal government or even the government of higher levels. Otherwise, Luohu would become an “isolated island” of health reform and no longer sustainable.

#### Health workers

Health workers at community health centres were highly motivated in Luohu’s health reform. General practitioners reported better benefits and more career development opportunities than before. Other health workers, such as public health practitioners and pharmacists, also had higher morale than before, as they could achieve personal fulfilment and had higher status. In general, health workers at community health centres felt supported and connected in the Luohu Hospital Group.

“Previously, doctors at community health centres usually asked us clinical questions through private connections. But now we are all in the Luohu Hospital Group. We have an obligation to support each other at work. It’s very convenient to communicate internally. (a doctor at Luohu Hospital)”

Although health workers at hospitals expressed solidarity with those at community health centres, they also conveyed their concerns about the reform. Before the reform, some health workers at Luohu Hospital (the largest district-level hospital in Luohu) were concerned about losing their benefits. In the meanwhile, health workers at smaller hospitals were afraid of losing hospital autonomy. After the reform, the purposes of reform were widely appreciated among workers. Salary of workers at all hospitals increased by about 20%, but variations in salaries existed across hospitals. Some specialists were not comfortable with the rising status of general practitioners in community health centres.

### Content

#### Principles and strategies

The principle of the reform in Luohu was letting residents in Luohu to have “less illness, fewer hospitalisations, less economic burden, and better care (pinyin: shao sheng bing, shao zhu yuan, shao fu dan, kan hao bing).” The main strategies were to create “a shared community of accountability, interest, and health (pinyin: ze ren gong tong ti, li yi gong tong ti, jian kang gong tong ti).” “A shared community of accountability” referred to the establishment of Luohu Hospital Group, which aimed at optimizing resource allocation across district-level hospitals and building a channel for strengthening community health centres. “A shared community of interest” was created by comprehensive financial arrangement to align provider incentives with the public interest. “A shared community of health” relied on primary care provided by family doctors at community health centres and other health promotion initiatives.

#### The Establishment of Luohu Hospital Group – A shared community of accountability

Luohu Hospital Group merged the resources of five district-level public hospitals and 23 public community health centres and founded single legal entity. The establishment of the Luohu Hospital Group fundamentally changed the governance structure of district-level public hospitals. The district government was no longer involved in operations management of district-level hospitals. Instead, the district government set up a council representing itself to make major decisions for the Luohu Hospital Group (e.g., the appointment of the chief director, major investments). Chaired by the Chief Executive, the council consisted of four government officials and six seasoned medical experts outside the Luohu Hospital Group. A supervisory board, consisting of representatives of the People’s Congress and the Committee of the People’s Political Consultative Conference, was also established to oversee the council and the Luohu Hospital Group.

“Four members of the council were government officials. …Hospitals are owned by the government. The council will not be like the board of directors of a stock corporation. Hospitals are government properties and should be controlled by the government.” (a government official).

Meanwhile, hospitals were granted greater managerial autonomy. Previously, directors and vice directors of public hospitals were appointed by the organisation department of the district party committee, and department chairs were appointed by the District Health and Family Planning Commission. After the reform, directors and vice directors of each hospital were named by the chief director and appointed by the council. Directors and vice directors of each hospital had the autonomy to decide department chairs and other administrative positions.

#### Comprehensive Financial Arrangement – A Shared Community of Interest

The government created incentives for the Luohu Hospital Group to strengthen primary care through reforming health insurance payment, increasing government funding, and adjusting prices.

First, while keeping current payment schemes (e.g., fee-for-service), the health insurance inpatient fund in Shenzhen created a global budget model for the Luohu Hospital Group, which was intended to promote greater care coordination, lower cost, and better outcomes. A single yearly budget was predicted by the historic inpatient spending of all contracted clients of the Luohu Hospital Group. The Luohu Hospital Group could earn the savings if their contracted clients spent less on inpatient care than the predicted budget. But they would not be financially penalized if they failed to meet the target. Notably, this budget included all inpatient care of contracted clients, including those outside the Luohu Hospital Group. As discussed before, health insurance plans in Shenzhen had a moderate restriction on patients’ choice of providers. Thus, it was hoped that the Luohu Hospital Group could attract and retain patients within Luohu Hospital Group through improved performance. However, it remained unknown whether Luohu’s health reform would slow the growth of health spending. The inpatient expenditure of contracted clients did not meet the target in the first year, and about 80% of costs incurred outside Luohu Hospital Group. Further initiatives in payment reform were still needed.

Second, the government significantly increased its funding for the Luohu Hospital Group and expected the group to strengthen primary care and act in the public interest accordingly. On average, government funding accounted for 20–30% of hospital revenue in all districts of Shenzhen. The government-funded public hospitals and community health centres based on the amount and quality of health services and subsidized health care facilities by purchasing services and block grants. The government further supported community health centres through funding basic public health services, training general practitioners, subsidizing specialists working at community health centres, and paying rents.

Moreover, Shenzhen adjusted prices of health services, removed the mark-up for medicines, and promoted bundled payments for some medical consumables, to contain the growth of health care costs.

#### Strengthening Primary Care – A Shared Community of Health

To expand contracted family doctor services was the main strategy to improve the services of community health centres. The goal was to provide family doctor services to all residents in Luohu. By the end of 2016, about half of residents in Luohu had a contracted family doctor, who was a general practitioner working with other providers (e.g., nurses, pharmacists, nutritionists) in a team. Residents’ decision as to whether to sign the contract with a family doctor team was completely voluntary.

The main barrier to strengthening primary care was the lack of qualified providers. Thus, while arranging financial, logistics, and technical support to build the capacity of community health centres, Luohu Hospital Group also made significant investments in the human resources for the community health centres. The group tried to increase the number of general practitioners through training and recruitment. To alleviate the shortage of qualified public health practitioners, experts from the centre for disease control and prevention were invited to train and work with community public health practitioners. Luohu Hospital Group also trained and mobilized about 1000 community health workers to assist general practitioners in outreaching residents and health education.

After the reform, many noted that the volume of services increased significantly at community health centres. Also, community health centres in Luohu ranked 1st in patients’ satisfaction among all districts in Shenzhen.

### Process

Luohu Hospital Group began with integration at the macro and meso levels and made unequal progress in integration at different levels (see Table 1).

**Table 1:** Summary of integration actions at the macro, meso and micro level.

Level of integration	Integration actions in Luohu Hospital Group

Macro level: System integration	Vertical: Integrated primary care and specialty care Horizontal: Integrated health care with public health services (e.g., health education, free vaccination, cancer screening, fall prevention) Integrated healthcare with social services (e.g., home care for disabled elders, community care in collaboration with daycare centers, institutional care incorporating health care with long-term care)
Meso level: Organisational integration	Vertical: Merged the resources of five district-level public hospitals and 23 public community health centers and founded single legal personhood Horizontal: Consolidated administrative and supporting departments of each hospital into six administrative centres and six supporting centres
Meso level: Professional integration	Vertical: Motivated specialists to train providers at community health centres Encouraged specialists to work part-time at community health centres Horizontal: Consolidated professional resources across hospitals
Micro level: Clinical integration	Vertical: Established a formal two-way referral system has been established Provided primary care doctors with timely decision support from specialists at hospitals Horizontal: Encouraged providers to integrate clinical pathways
Linking the macro, meso and micro level: Functional integration	Shared key support functions, including strategic planning, human resources, financial management, information system, and quality control.
Linking the macro, meso and micro level: Normative integration	Guided by a “health-centered” rather than a disease-centred perspective Shared the goals of “less illness, fewer hospitalisations, less burden, and better care”

#### System integration – integrating health care with public health and social services

Community health centres had been directly funded by the government for providing basic public health services (e.g., physical exams, vaccination, health education, chronic disease management) before the reform in Luohu. To expand contracted family doctor services, community health centres often engaged residents first through basic public health services, especially free physical exams for people aged 60 or over. Then, family doctors and their team gradually built connections with patients to deliver primary health care, arrange referrals, and manage chronic diseases. To help patients monitor and manage chronic diseases, one community health centre even experimented with a commercial health information system which could measure patients’ vital signs at home and share the information with doctors online.

Luohu Hospital Group also provided a series of preventive services in addition to basic public health services. Examples included health education, providing fall-prevention grab bars for elders, free vaccination to prevent pneumonia, and cancer screening.

Regarding social services, the Luohu Hospital Group experimented with integrating health care and long-term care for elderly patients. Community health centres provided nursing, rehabilitation, and palliative services at the home of contracted elderly patients. Community health centres also collaborated with daycare centres to provide community care for disabled elders. Moreover, the Luohu Hospital Group had a geriatric hospital to provide institutional care incorporating health care and long-term care.

“Many residents reached us to set up a home bed. Our family doctors will evaluate the patient using the electronic health record system and do a home visit to assess the home environment and service needs. … Previously, many elders with chronic conditions, especially those with difficulties in activities of daily living or cognitive problems, had no one to care for them. Now we have family doctors to visit them on Monday, Wednesday, and Friday every week, to provide health education and help manage their medications.” (a hospital manager).

#### Organisational integration

**Figure 2:**
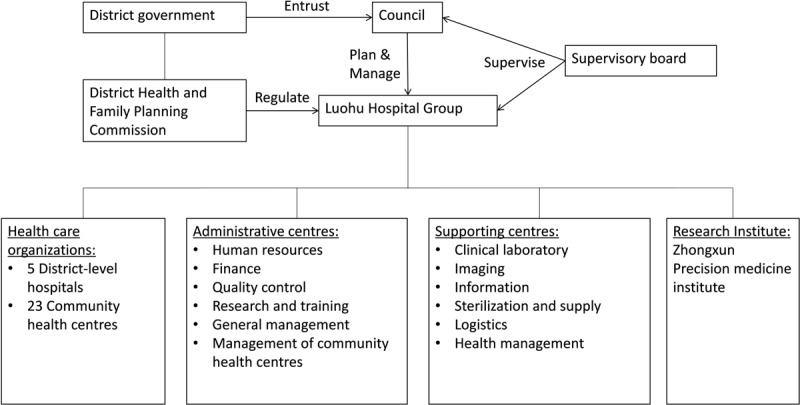
The governance and organisational structure of Luohu Hospital Group.

The Luohu Hospital Group consolidated administrative departments of each hospital into six administrative centres (e.g., the centre for human resources, the centre for community health) and merged labs, radiology, and logistics departments into six supporting centres (e.g., the centre for imaging, the centre for clinical laboratory) (see Figure 2). There was a consensus that the centre for imaging was particularly valuable and should be scaled up in other parts of China. All radiologists were working in the centre for imaging, which improved the efficiency and quality of radiology services. Meanwhile, technicians were sent to hospitals and community health centres, making imaging services more accessible to residents. Images were easily shared within a single electronic health record system. The Luohu Hospital Group envisioned the centre for imaging a node of a larger radiology network: the centre was connected to more than 100 hospitals in other parts of China to assist local providers; radiologists in Luohu can also reach radiologists all over the world to diagnose complicated cases. In addition, the centre for clinical laboratory was considered beneficial in cost containment and quality control. Previously, hospitals tended to provide only routine tests for cost considerations. The establishment of the centre for clinical laboratory made both routine tests and non-routine tests available in all hospitals. But some noted that the centre moderately increased waiting time for routine tests for logistics reasons, as specimens should be sent to centre and could not be examined onsite.

Though rewarding, many considered establishing a single legal entity was a particularly difficult process due to the concerns and objections from health workers and hospital leaders. To obtain support from hospital leaders, the director of the Health and Family Planning Commission talked to leaders of each hospital to convince them that the Luohu Hospital Group intended to achieve joint development. While establishing the Luohu Hospital Group, the government changed more than 40 directors and vice directors of hospitals. Dramatic personnel changes also occurred at the level of department chairs. Even after the reform, there were mixed perceptions about the adjustments in the governance structure. First, having a single legal entity made it less convenient for hospitals to collaborate with institutions outside the group. Moreover, to control operating costs and improve efficiency, leaders of the Luohu Hospital Group were often the director of hospitals. For instance, the chief director was also the director of the Luohu Hospital. But some argued that such an arrangement might result in conflicts of interest. Some also noted that to build such an integrated network was feasible in Luohu because district-level hospitals in Luohu were close to each other geographically, and community health centres were all run by district-level hospitals. Thus, many considered that, though crucial, establishing single legal entity of a hospital group might be even more difficult in other places.

#### Professional integration

Horizontally, the Luohu Hospital Group rearranged professional resources to differentiate and strengthen each hospital’s specialities. For instance, the Luohu Hospital planned to close its traditional Chinese medicine department and sent all traditional Chinese medicine doctors to the Luohu Traditional Chinese Medicine Hospital. But some hospitals were concerned about losing key departments. According to the current hospital accreditation standard in China, to be accredited as a tertiary hospital required the hospital to have a certain set of clinical departments.

Vertically, the Luohu Hospital Group encouraged specialists to work part-time at community health centres. For senior doctors, Luohu Hospital Group subsidized them if they provided outpatient services at community health centres, while for junior doctors, working at community health centres was required for their promotion. For instance, the department of gastroenterology of the Luohu Hospital sent out physicians to a community health centre every workday and provided gastroscopy and colonoscopy services there daily. Before the reform, the department of gastroenterology admitted patients with common illnesses to maintain the volume of services. After the reform, they saw patients with common illness at the community health centre and only admit those who needed hospitalisation. They also provided gastroscopy and colonoscopy services in the community to screen early cancer. However, not all departments were motivated to build connections with community health centres. Clinical departments primarily dealing with chronic diseases (e.g., respiratory department) were more interested in training and collaborating with community health centres, compared to departments mainly responsible for acute episodes (e.g., the department of surgery).

#### Clinical integration

Horizontally, Luohu Hospital Group consolidated clinical resources across hospitals. Clinical collaborations across hospitals dramatically increased after the reform. Providers of the same speciality tended to collaborate with providers from other hospitals, rather than compete against each other. For instance, the Luohu Hospital and the Luohu Maternal and Child Health Centre collaborated in providing In Vitro Fertilisation which required close observation before the procedure. Such arrangement shortened waiting time and improved patients’ experiences.

Vertically, a formal two-way referral system has been established between community health centres and hospitals. The priority of scheduling hospital services was given to contracted clients with referrals from general practitioners. During the patient care process, general practitioners could receive timely decision support from specialists within Luohu Hospital Group. Also, patients could access more medicines and more comprehensive clinical laboratory and radiology services at community health centres than before.

#### Functional integration and normative integration

Health care facilities within Luohu Hospital Group shared key support functions, including but not limited to information system, financial management, and human resources. All facilities within the group used the same electronic health record system, which was essential for the vertical and horizontal integration of clinical processes. The group also managed to consolidate the financial system of each institution, despite considerable technical challenges. However, the rearrangement of human resources were complicated by the quota system, as discussed before. With the quota system, employees might not move as needed to another institution even within the group.

The Luohu model emphasized a “health-centred” rather than a disease-centred perspective. Such value was gradually embraced by providers in their daily clinical practice. For instance, many doctors considered seeing patients at community health centres as an opportunity to reach out to patients, improve patients’ experiences, provide proactive and preventive care, and train providers at the grassroots level, rather than a task required by the hospital.

“We did cancer screening. You must go to the community to develop your speciality and educate patients. If you do not go there, patients won’t know you are an expert. We hospitals cannot advertise ourselves. We just go to patients and communicate with them. Let them decide whether to trust you.” (a nurse).

## Discussion

This study was the first qualitative study examining the context, actors, content, and process of founding the Luohu Hospital Group. The reform in Luohu took place in the midst of the comprehensive health reform in Shenzhen and within a competitive health care market. The district government initiated the reform, with the support of the municipal government and health care providers. The reform aimed at strengthening primary care and care coordination, improving the quality and efficiency of health care delivery, and promoting population health. Formal linkages and unified information system were set up to coordinate services between hospitals and community health centres. Increased government funding and a global budget model were arranged to align provider incentives. The establishment of the Luohu Hospital Group transformed the originally fragmented delivery system into a tightly ISDN.

The reform in Luohu echoed with integrated care efforts in both high-income countries and low- and middle-income countries. The Luohu model emphasized the role of primary care providers to coordinate services across the continuum of care, which was the focus of the patient-centred medical homes in the United States. The Luohu Hospital Group was also similar to an accountable care organization (ACO) in the United States, which aimed at the alignment of incentives and accountability for providers [37,38]. The Luohu model was also similar to some initiatives in building integrated care systems (ICS) in the United Kingdom (UK), in which all the participating organisations share accountability for health care and ideally social services provided to a defined population, within a capped budget. However, Luohu Hospital Group was not paid according to quality measures or health needs of a defined population. Notably, many elements (e.g., integration of health care and social services, emphasis on collective accountability and risk-sharing across the system) of the Luohu model was not that innovative when compared with initiatives in the US and the UK. But in the context of China, such integration efforts were encouraging, though still at an early stage. Moreover, the integration of health care with vertically funded public health services in Luohu was similar to many integrated care programs in low- and middle-income countries, many of which were faced with the tension between strengthening primary health care systems and expanding health-need specific programs funded by donors, such as those for HIV, malaria, and family planning [39]. Basic public health services in China was funded jointly by all levels of government, and these programs were rarely coordinated with health care. In the Luohu model, community health centres used basic public health services as an entry point of health care to build trust in the community.

As discussed in previous literature [24,25,26,27,28], the Luohu model generated valuable lessons for integrated care initiatives in China, particularly the establishment of ISDN. ISDN were faced with several shared challenges to achieving integrated care [40,41]. First, few ISDN achieved organisational integration. Organisational integration was a particularly demanding task in urban China due to the governance structure of public hospitals. To realise organisational integration, the reform in Luohu combined the formation of an ISDN with public hospital reform, which was the prerequisite of restructuring hospitals within the Luohu Hospital Group (e.g., consolidating supporting resources). Second, a shared electronic health record system was also a crucial tool to link facilities in the group. Third, providers within an ISDN were often not incentivized to integrate care at the clinical level. The reform in Luohu coordinated delivery system reform with payment reform. Comprehensive government investment was in place to motivate primary care providers at the grassroots level and incentivize hospitals to support community health centres. A global budget for the whole group was a crucial strategy to harmonize the interest across providers. Fourth, primary care facilities within anISDN often lacked the capacity to provide high-quality health care and were not trusted by local residents. Luohu Hospital Group prioritized the capacity building of community health centres to boost their health care quality. To build trust, providers at community health centres outreached local residents proactively, and Luohu Hospital Group did not restrict contracted clients’ choice for care.

Considering the growing interest in scaling up the Luohu model in China [29,30], the context and primary actors of the reform in Luohu should also be noted. Backed by Shenzhen’s strong economy, the city-level health reform in Shenzhen created an excellent policy environment for the reform in Luohu. The universal health insurance coverage and public hospital reform in Shenzhen was the foundation for the establishment of Luohu Hospital Group. Also, born in a competitive health care market, the Luohu Hospital Group would not become a monopoly provider if patients could “vote with their feet.” Such a market context might not be found in many other areas in China. The primary actor in the reform was the district government in Luohu. China’s health reform was a mix of top-down and bottom-up processes. On the one hand, local governments often experimented with reform models following the guidelines of the central government. On the other hand, the central government endorsed successful models and scaled them up nationally. Under most circumstances, the district government implemented the reform initiated by the municipal government or the provincial government. But in Luohu, the district government was the primary actor of the reform. Compared to reforms initiated by higher levels of government, the reform in Luohu adopted a bottom-up approach, which fitted the local health care market and policy environment. The strong leadership of the district government was the key to success in the reform of Luohu. The health reform was among top priorities of the district government. Leaders of the reform were resourceful and skilful in engaging different actors and mobilize health workers in the implementation process. But the future of the Luohu model might be shaped by the interaction between the district government and higher levels of government. Many challenges for the Luohu Hospital Group (e.g., the quota system, coordination with city-level hospitals) could only be solved in a top-down process with the support from higher levels of government.

The findings should be interpreted with caution. First, this study did not include patients’ experiences and perspectives. In another study, we investigated patient satisfaction using a cross-sectional survey. With a response rate of 95%, a total of 936 patients were surveyed at three hospitals and 23 community health centres within the Luohu Hospital Group [28]. The results suggested that the majority of patients were satisfied with the services provided by family doctors and community health centres. Patients also reported increased willingness to seek health care at community health centres after the establishment of Luohu Hospital Group. Second, this study did not include the perspectives from non-government providers in Louhu. Furthermore, this study was conducted during the process of the reform in Luohu, many important outcomes, such as quality of care, were not covered in the interviews and focus groups, and future monitoring was warranted.

## Conclusions

The reform in Luohu took place in a competitive health care market, based on the comprehensive health reform in Shenzhen. Luohu Hospital Group was established under the strong leadership of the district government. The reform adopted comprehensive strategies to strengthen primary care and care coordination, improve the quality and efficiency of health care delivery, and promote population health. The reform achieved a high level of organisational integration but was still in the process of fulfilling professional and clinical integration. Though valuable lessons have been generated, the reform and its outcomes require ongoing monitoring.
